# Distribution and Localised Effects of the Invasive Ascidian *Didemnum perlucidum* (Monniot 1983) in an Urban Estuary

**DOI:** 10.1371/journal.pone.0154201

**Published:** 2016-05-04

**Authors:** Tiffany Schenk Simpson, Thomas Wernberg, Justin I. McDonald

**Affiliations:** 1 School of Plant Biology and UWA Oceans Institute, University of Western Australia, Crawley 6009, Perth, Western Australia, Australia; 2 Biodiversity and Biosecurity, Western Australia Fisheries and Marine Research Laboratories, Perth, Western Australia, Australia; Seagrass Ecosystem Research Group, Swansea University, UNITED KINGDOM

## Abstract

Didemnid ascidians are notorious marine invaders, fouling infrastructure in many ecosystems globally. However, there have been few reports of direct interactions with native species in their natural environment. The invasive colonial ascidian *Didemnum perlucidum* was discovered in the Swan River estuary (Western Australia) growing on the native seagrass *Halophila ovalis*. Given the known effects of other related *Didemnum* species it was expected that *D*. *perlucidum* could adversely affect the seagrass, with possible flow on effects to the rest of the ecosystem. This study aimed to document the distribution and abundance of *D*. *perlucidum* in the estuary, and to determine whether this species had a negative impact on *H*. *ovalis* or associated flora and fauna. *D*. *perlucidum* was largely present near areas of infrastructure, particularly mooring buoys, suggesting these were the source of *D*. *perlucidum* recruits on the seagrasses. It showed a clear seasonal pattern in abundance, with highly variable cover and colony size. *D*. *perlucidum* had a measurable effect on *H*. *ovalis*, with colonies enveloping all plant tissue, likely restricting the photosynthetic ability of individual leaves and total plant biomass. There were also significantly less seagrass-associated mud snails (*Batillaria australis*) where *D*. *perlucidum* cover was high. These results demonstrate the ability of invasive ascidians to colonise and affect native seagrasses and associated biota. Seagrasses are pivotal to the ecological function of many urban estuaries world-wide. Biodiversity in these systems is already vulnerable to multiple stressors from human activities but the potential stress of fouling ascidians may pose an additional and increasing threat in the future.

## Introduction

The introduction of non-indigenous species (NIS) that become invasive is an environmental challenge affecting the world’s oceans and coastal ecosystems, including estuaries [1, 2, 3]. The effect is accelerating rapidly as vectors for introduction continue to increase [4, 5].

Estuaries are semi-enclosed coastal water bodies with a free connection to the open ocean and within which seawater is measurably diluted with fresh water from land drainage [6]. These areas constitute transition zones between land and sea, creating some of the most biologically productive areas on Earth [2, 7]. Seagrass meadows are a dominant habitat in many estuaries where they offer a variety of important ecosystem services to coastal regions such as increasing habitat complexity, stabilising sediments, filtering runoff, providing habitat for other plants and animals, and carbon sequestration [2, 8, 9, 10, 11]. However, these transition zones are often focal points for multiple human activities, concentrating anthropogenic influences such as sedimentation, nutrients and pollution into localised areas. Stress on the natural ecosystem combined with multiple vectors for introduction conditions estuaries to become sinks for new opportunistic species [9, 11, 12].

At least 56 non-indigenous species have been documented in seagrass ecosystems worldwide, of which 64% have been demonstrated or inferred to have negative effects [2, 3]. Negative effects include alterations to energy flow and dynamics of benthic communities [13], decreased seagrass photosynthesis and growth [3], reduction in species diversity, shifts in trophic organisation, infiltration of pathogens and alteration of habitats [7].

*Didemnum perlucidum* is a colonial ascidian believed to be native to tropical Indo-Pacific waters [14]. There is strong evidence suggesting that *D*. *perlucidum* may be a very successful invader [15, 16]. Like many ascidian species it has life history traits that favour invasion including rapid growth, high fecundity and multiple reproductive strategies, including the ability to regenerate from fragments [14, 17, 18, 19, 20].

*Didemnum perlucidum* was first documented in Western Australia in 2010 growing on settlement panels and jetty pylons in the lower reaches of the Swan River estuary. Colonies were observed overgrowing other fouling organisms and formed continuous mats that covered up to 50% of pylon surfaces [21]. Since then, *D*. *perlucidum* has been confirmed in several locations along the coast of WA on artificial structures from Broome to Esperance [16, 22], spanning a latitudinal range of about 16° from tropical to temperate waters. Due to its potential negative impacts *D*. *perlucidum* has been added to the Western Australian Prevention List for Introduced Marine Pests [23] as well as the United States National Exotic Marine and Estuarine Species Information System [24]. Observations in the Swan River estuary between 2013 and 2015 have shown that *D*. *perlucidum* was also living on leaves of the native seagrass *Halophila ovalis* (McDonald personal observation), which has not previously been documented.

Ascidian fouling has been implicated as a probable driver of seagrass decline in some ecosystems, often causing negative effects on seagrass photosynthesis and growth [11, 25], likely through smothering the plants [26, 27]. In New England (USA), *Didemnum vexillum* spread to eelgrass (*Zostera marina*) habitats, contributing to a loss of eelgrass as well as negative impacts on a commercial scallop fishery [27]. Ascidian fouling of eelgrass beds has demonstrated deleterious effects including reduced plant growth, decreased light attenuation and decreased chlorophyll *a* concentrations [26]. Fouled plants may also collapse under the weight of the ascidians and break away, contributing to the decline of the seagrass canopy as well as furthering spread of the invasive ascidians [3].

The Swan River-Cockburn Sound estuarine ecosystem is located within the city of Perth, capital of the state of Western Australia. It supports considerable biodiversity and is one of the most commercially and recreationally exploited coastal ecosystems in Australia, supporting activities contributing >$40 billion to the national economy each year [28]. Seagrasses are an important component of this aquatic ecosystem because of their role as primary producers, provision of habitat, sediment stabilisation and as a food source [2]. In addition to carbon, the oxygen produced is important in creating oxic conditions for other animals around the water-sediment interface [10]. The dominant seagrass in the Swan River, *Halophila ovalis*, is a highly productive species, with an estimated net primary production of 500g C m^-2^ year^-1^ [29]. *H*. *ovalis* meadows are estimated to cover about 20–25% of the total estuary benthos, with the Department of Water estimating coverage of about 403 hectares in 2011 [10]. However, as is the global pattern, *H*. *ovalis* has been declining in the Swan River, primarily due to human activities and stressors including increased temperature and sedimentation, excessive nutrient runoff, seaweed proliferation and invasion of NIS [3, 29, 30]. The introduction of the invasive colonial ascidian *D*. *perlucidum* may be an additional threat to the health and abundance of the native seagrass *H*. *ovalis* which could have ‘flow-on’ effects to the rest of the ecosystem. Consequently, it is important to identify whether there is an effect of *D*. *perlucidum* on *H*. *ovalis*, the scale of the effects and whether there are likely irreversible large-scale future impacts. The aims of this study were to 1) quantify the distribution and abundance of *D*. *perlucidum* on natural and artificial substrates in the Swan River under different seasonal conditions, and to 2) determine whether the colonies of *D*. *perlucidum* could be causing negative effects on *Halophila ovalis* and associated flora and fauna, particularly *Batillaria australis*.

## Methods

### Distribution and temporal variation in abundance of *Didemnum perlucidum*

**Fig 1:**
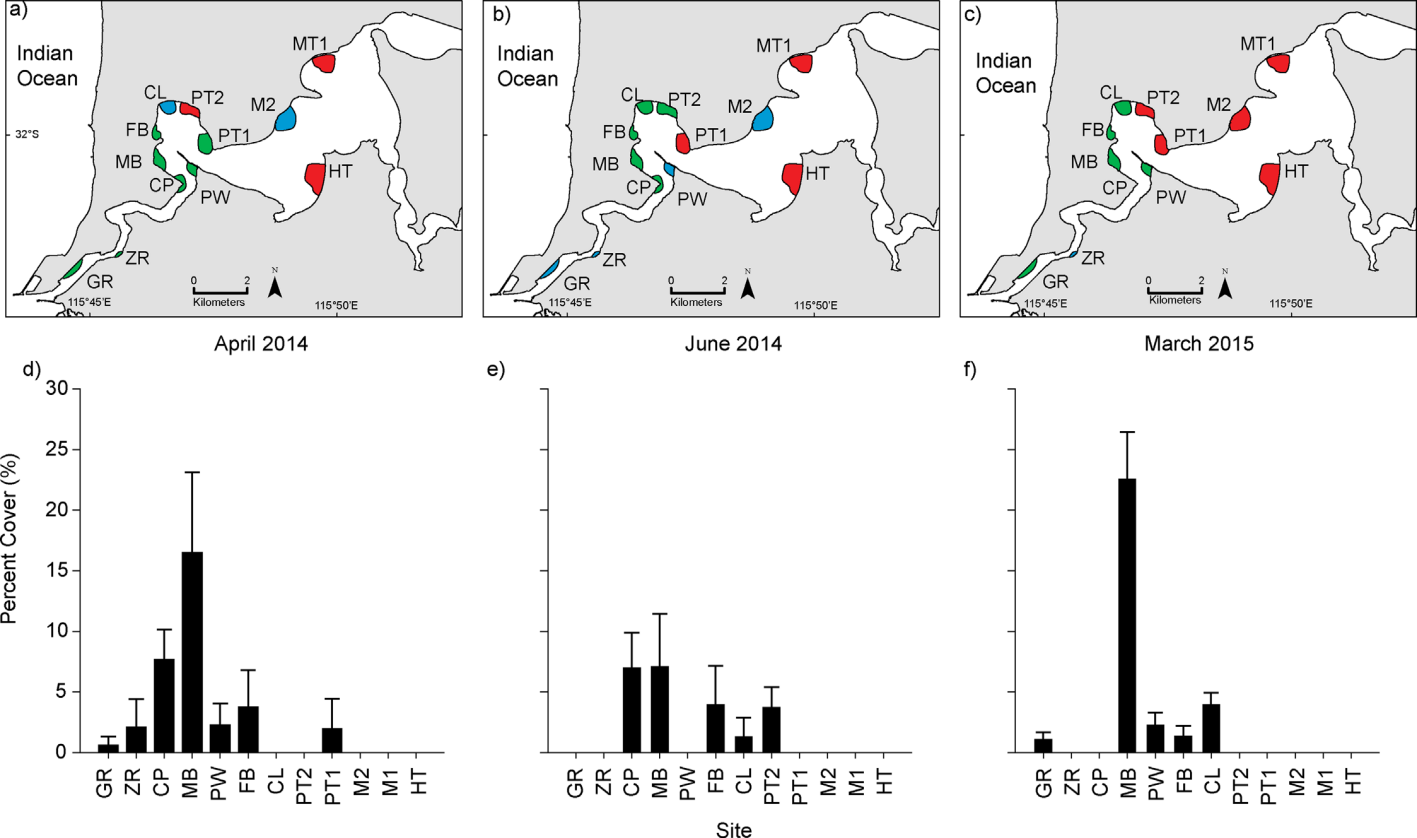
**a-c)** Map of the Swan River showing the 12 sites sampled in April and June 2014 and March 2015. Green indicates *Didemnum perlucidum* growing on seagrass and artificial substrates, blue indicates *D*. *perlucidum* growing only on artificial substrates and not seagrass, red indicates no presence of *D*. *perlucidum*. **d-f)** Percent cover plus standard error of *D*. *perlucidum* on seagrass at each site in April, June and March. Sites are along the horizontal axis and run from the lower estuary upstream towards Perth CBD. (Site name abbreviations: GR- Gilbert Fraser Reserve, ZR–Zephyr Café, CP–Chidley Point, MB–Mosman Bay, PW–Point Walter, FB–Freshwater Bay, CL–Claremont, PT–Point Resolution, M–Matilda Bay, HT–Heathcote Reserve)

To establish the distribution and temporal variation in abundance of *D*. *perlucidum* growing on seagrasses and infrastructure, 12 sites were surveyed across the lower reaches of the Swan River between the river mouth and the Narrows Bridge, about 15 km upstream (Fig 1). Permission for surveying the sites was granted by a permit from the Swan River Trust and all flora and fauna sample collections were supported by permits from the WA Department of Parks and Wildlife. Snorkelers and SCUBA divers surveyed seagrass meadows, moorings, and jetty pylons in April, June and August 2014 to document the seasonal peak through to the decline in *D*. *perlucidum* abundance as well as a follow-up in March 2015 to confirm the reappearance the following year. Within each site, 3 x 60m parallel transects were surveyed perpendicular to the shore. Six random 0.25x0.25m quadrats were observed and photographed at 20, 40 and 60m along each transect. Within the quadrats, percent cover of seagrasses and *D*. *perlucidum* was estimated. Observations of cover for seagrasses and *D*. *perlucidum* were classed into categories of 0, 1–10%, 11–25%, 26–50%, 51–75%, 76–90% and 91–100%. The midpoint of each of these cover categories was used as the reported value, to allow for calculating average percent cover and standard deviation [10]. Observations and photographs were recorded at adjacent moorings and jetty pylons to determine presence/absence of *D*. *perlucidum* on artificial structures adjacent to seagrass meadows.

Statistical analyses were performed using Primer 6 software [31] with the PERMANOVA add-on package [32]. Differences in cover of *D*. *perlucidum* among sites (random factor), times of the year (random factor) and transects (random factor nested in site) were tested by analysis of variance by permutation (PERMANOVA). Data was Log(x+1) transformed with Euclidean Distance resemblance.

For each site a range of factors were recorded to determine if any environmental conditions correlated with the distribution of *Didemnum perlucidum*. Measurements of temperature and salinity were taken at each site using a YSI multimeter probe. Baardseth’s index was used as a proxy for water movement due to wave exposure at each site. This was calculated by counting the number of 10° sectors with a 3.75 km open fetch [33, 34]. Thus, the exposure scale ranged from 0 = fully protected to 36 = fully exposed. Measurements were made on a marine chart with a 1:25,000 scale. This chart was also used to measure the distance of each site to the nearest jetty and to the river mouth. The number of moorings present within 100m by 100m around the measured transects was also recorded. Distance-based redundancy analysis (DISTLM) was used to identify the subset of environmental factors that best explained the distribution of *D*. *perlucidum* and identify the correlation of each factor. For this analysis environmental data were normalised and D. *perlucidum* cover data was square root transformed with Bray Curtis resemblance plus a dummy variable to reduce the influence of many zero values.

A permanent 10m x 10m quadrat was established at Point Walter from April 2013 to March 2014 to further assess the growth and spread of *D*. *perlucidum* colonies over time. The entire quadrat was photographed, (100 1m^2^ photos) every month. Image J software was used to measure the area of all emergent colonies. To test for differences in colony size within each quadrat from month to month we performed a repeated measures analysis of variance (ANOVA).

### Effects on seagrass

Within five of the seagrass sites, (ZR, RB, MB, FB and PT, Fig 1), biomass samples were collected using randomly placed 10cm x10cm quadrats. Three samples of *Halophila ovalis* only and three samples of *H*. *ovalis* with *Didemnum perlucidum* were collected (total n = 30). The *D*. *perlucidum* was carefully separated from the *H*. *ovalis* and each sample was dried for 24 hours at 40°C and then weighed. A PERMANOVA (Euclidian Distance, 9999 permutations) was performed to test for significant differences in seagrass biomass between sites and samples with and without *D*. *perlucidum*.

Photosynthetic response of *Halophila ovalis* leaves that had been covered with *Didemnum perlucidum* colonies were compared in the laboratory to leaves without *D*. *perlucidum*. Samples were collected from Freshwater Bay and Mosman Bay in February 2015 in approximately 2 metres depth. Five *D*. *perlucidum* colonies, along with five adjacent unaffected samples of *H*. *ovalis*, were collected from each site (total n = 20). Leaves from within the *D*. *perlucidum* colonies were then separated from the tunicate tissue. Pulse amplitude modulated (PAM) chlorophyll fluorescence parameters were measured from the middle of the leaf using a Mini-PAM (Walz, Germany). Maximum quantum yield (F_v_/F_m_) was measured, as this is frequently used as an indicator of photo-physiological stress to the PS2 complex. Data were downloaded and analysed using the WinControl-3 software. All leaves were placed in plastic ‘leaf clips’ and dark adapted for 10 minutes. Rapid light curves were produced using an incremental sequence of actinic illumination with seven discrete irradiance steps (0, 45, 90, 180, 300, 500, 700 μmol quanta m^-2^s^-1^). The maximum electron transfer rate (ETR_max_) and photosynthetic efficiency (α) were calculated by fitting the rapid light curve data to an exponential function [35].

Pigment levels were measured by cutting a 1cm diameter disks from each leaf, measuring the wet weight and soaking them overnight in 3ml of 100% acetone. The following day, the remaining leaf tissue was removed and the acetone was mixed with 1 ml distilled water and 1ml methanol. Solutions were centrifuged for 3 minutes at 1500rpm and kept on ice. The top 3ml was measured using a spectrophotometer at 664nm wavelength to determine the concentration of chlorophyll *a* [36].

Photosynthetic activity and pigment concentrations can only be measured on leaves that are still alive. Consequently, this produced an overestimation of the health of the leaves within the *D*. *perlucidum* colony, as most of the leaves were dead. Therefore an index of the health of leaves within and without *D*. *perlucidum* colonies was estimated following the scale: 1 = alive (green), 2 = alive with necrotic patches (green with brown spots), 3 = dead (various shades of brown). This index was used to qualify the photosynthetic and pigment results.

### Effects on *Batillaria australis*

*Battilaria australis* is a mud snail which is currently the most abundant macroinvertebrate in the Swan River [37]. These snails were sieved from sediment cores with and without *Didemnum perlucidum* colonies. Sediment cores (90mm diameter, 60mm depth) were collected from 3 sites (ZR, MB and FB) in April and June 2014 and March 2015. Within each site, 6 cores were collected, 3 containing a *D*. *perlucidum* colony and 3 without (total n = 18). *B australis* individuals were counted in each core and analysed by PERMANOVA (data Logx+1 transformed, Euclidean Distance resemblance) following a design of treatment (with or without *D*. *perlucidum*) (fixed), time of year (random) and site (random).

## Results

### Distribution and monitoring of *D*. *perlucidum*

**Fig 2:**
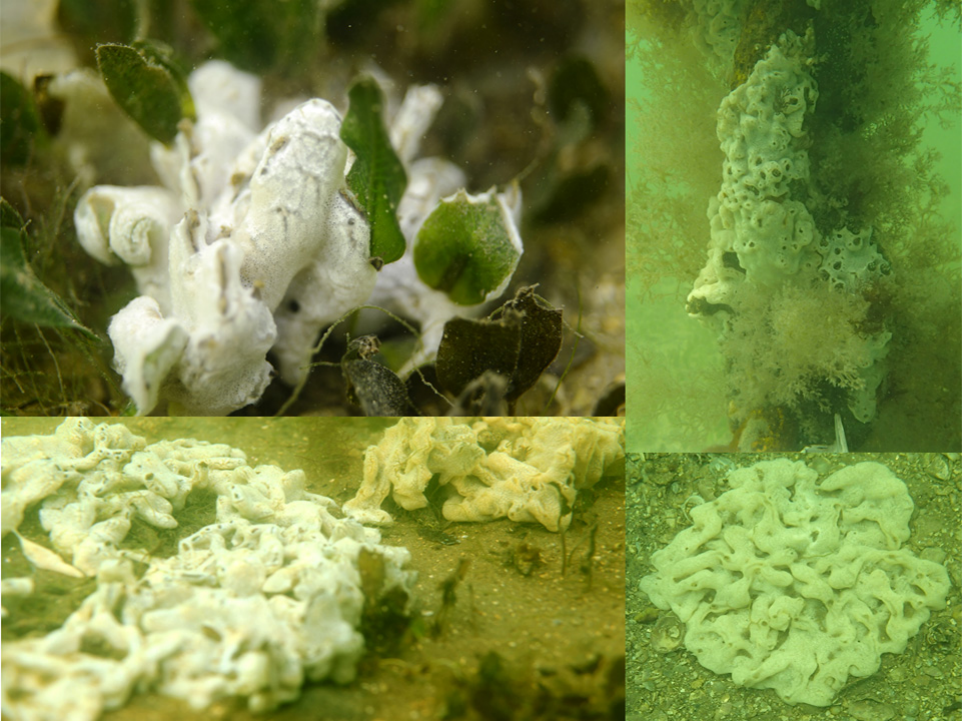
*Didemnum perlucidum* on seagrass, a navigation marker and bare substratum.

In April 2014, *D*. *perlucidum* was observed at 86% of the sites surveyed in the Swan River (S1 Table). Of those, 75% had *D*. *perlucidum* growing on seagrass, primarily on *Halophila ovalis* but in Rocky Bay, where other seagrasses are present, it was also growing on *Zostera marina*. Occasionally it was even seen growing on the macroalgae *Gracilaria comosa*, *Cystoseira sp*. and *Chaetomorpha sp*. Where *D*. *perlucidum* was associated with *H*. *ovalis* it was observed on individual seagrass blades but more often was spread across numerous blades, forming mats up to 30cm in diameter (~900 cm2). Most observations of *D*. *perlucidum* on seagrass were found within 10s of meters of colonies growing on artificial structures such as jetties and mooring lines. Colonies were recorded from both natural and artificial substrates in the Swan River between 2m and 10m depth (Fig 2). In June, *D*. *perlucidum* was only present on seagrass at 58% of the observed sites but was still widespread on artificial surfaces. In August, there was no observable *D*. *perlucidum* on either seagrass or artificial structures at any of the sites. In March 2015, *D*. *perlucidum* had returned on seagrass at 42% of the sites and artificial structures in 86% of the sites (Fig 1).

The cover of *Didemnum perlucidum* varied considerably over time (Fig 1 and Table 1). In April, mean (± SE) cover across the estuary was 3% (±1.4) with a maximum mean cover of 17% (±6.6) at Mosman Bay. In June, mean percent cover was 2% (±0.8) with a maximum of 7% (±4.4) at Mosman Bay. By August and through to the following year there was no *D*. *perlucidum* visibly present at any of the sites being monitored. By March 2015 it was present again, with a mean cover of 2.6% (±1.9). Again, the highest cover was observed in Mosman Bay, peaking at 22.6% (±3.9).

**Table 1:** Analysis of Variance by Permutaion (PERMANOVA) testing for differences in percent cover of *D*. *perlucidum* among sites (random factor), times of the year (random factor) and transects (random factor nested in site). The analyses were based on Euclidean Distances calculated from Log(x+1) transformed data.

Source	df	MS	Pseudo-F	P(perm)	Unique Perms
**Time of Year**	2	6.981	4.83	0.016	9946
**Site**	11	3.535	2.10	0.014*	9912
**Transect(Site)**	24	0.379	1.28	0.223	9912
**Time x Site**	22	1.455	4.94	<0.001*	9914
**Time x Transect(Site)**	48	0.294	0.55	0.995	9863
**Residual**	227	0.540			

Within the permanent quadrats, at the beginning of the study in April, *D*. *perlucidum* colonies had an average size of 106cm^2^ ± 7.3 SE with a range of 6.8 to 853cm^2^. Colony size contracted significantly by May and continued to decrease each month through the winter. By August there were no noticeable colonies. We continued to monitor the permanent quadrat until April 2014. In that time, *D*. *perlucidum* never returned.

Though *D*. *perlucidum* was not present within the permanent quadrat in April 2014, it was present about 100m away on *Halophila ovalis* clustered around a navigation marker. It was also present on the marker itself. Colonies within a 20m radius of the marker were measured to compare to the size of colonies from the previous year (n = 40). The average area of the colony was 33.1cm^2^ ± 6.6 and ranged from 1 to 196 cm^2^, demonstrating the extreme variability of colony sizes at that time.

Of the abiotic variables measured throughout the study, the multiple regression model (DistLM) found the number of moorings within a site to explain the highest proportion of variation in *D*. *perlucidum* abundance, although this was only about 16% of the variation in *D*. *perlucidum* cover across the range of sites and seasons. Salinity (14–39.4ppt, S1 Fig) contributed the second-most, explaining 10% of variation in cover (Table 2A and 2B). Water temperature ranged from 13.1° to 27°C (S1 Fig) and was expected to be highly correlated with abundance and distribution of *D*. *perlucidum* based on previous observations but in this model it was not significant. Maximum depth correlated significantly but only explained very little variation in distribution. Other factors including wave exposure and distance to jetties were not significantly related to the distribution and abundance (Table 2A).

**Table 2:** a) DistLM marginal test of contribution of factors to *D*. *perlucidum* distribution and b) model selections (Biological data square root transformed, Bray Curtis similarity, predictor environmental variables normalised, AICc selection criteria, stepwise selection procedure).

a) Marginal Tests						
Variable	SS(trace)	Pseudo-F		P	Prop.	
Temperature	1691	2.92		0.092	0.06	
Salinity	3886	7.34		0.008	0.14	
Max Depth (m)	2329	4.12		0.038	0.09	
Dist. to closest jetty (m)	110	0.18		0.734	0.004	
Moorings present	4280	8.22		0.006	0.16	
Wave exposure	728	1.21		0.273	0.03	
b) Model Selections						
Variable	AICc	SS(trace)	Pseudo-F	P	Prop.	Cumul.
Moorings present	290	4280	8.22	0.0048	0.16	0.16
Salinity	287	2594	5.89	0.02	0.1	0.25
Max depth	285	1841	4.18	0.04	0.07	0.32
Wave exposure	285	1038	2.44	0.12	0.03	0.35

### Effects on seagrass

**Fig 3:**
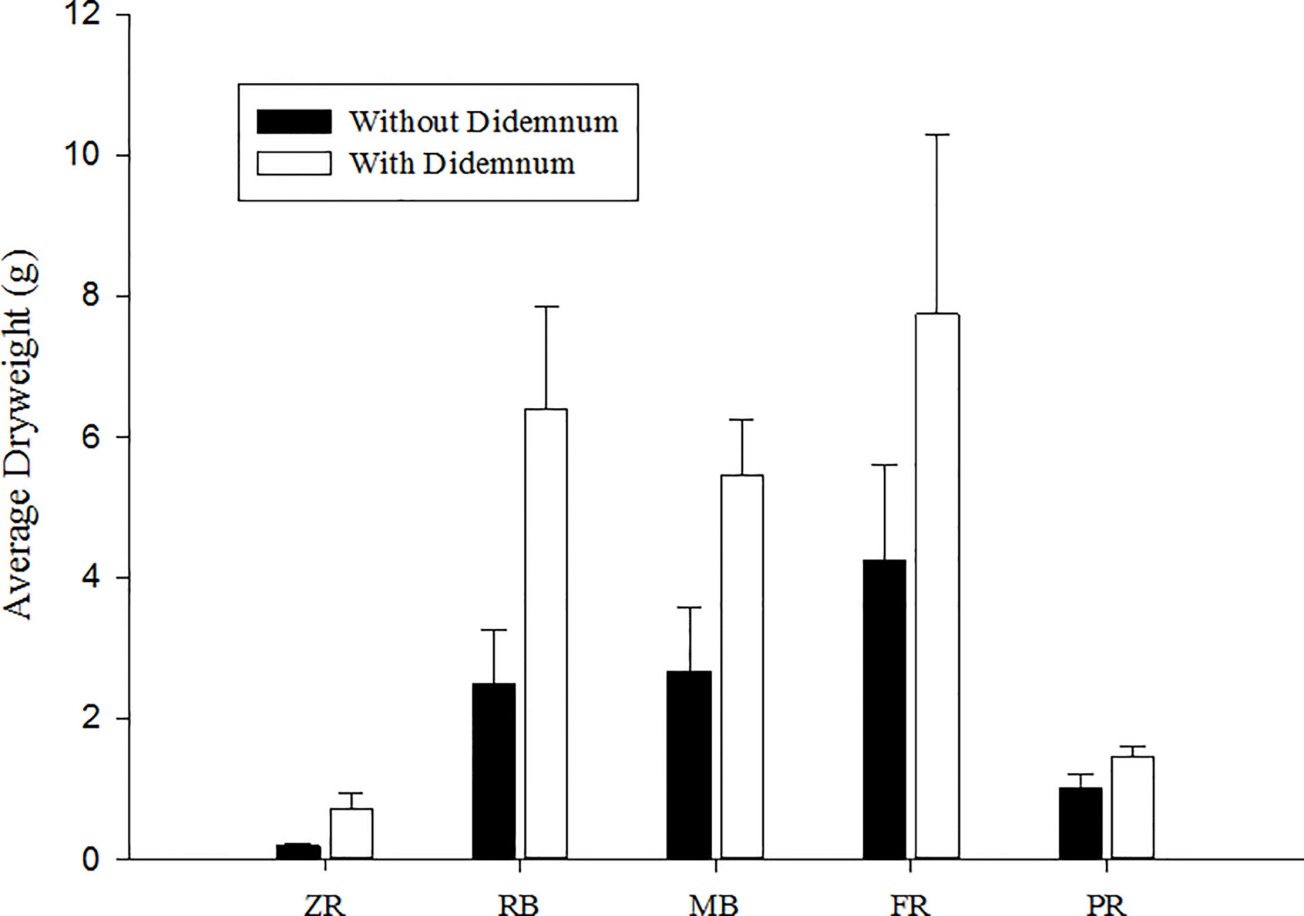
Dry weight biomass of *Halophila ovalis* with and without *D*. *perlucidum*. Colonies sampled and pooled from 5 sites in April and June 2014 and March 2015.

*Halophila ovalis* biomass was significantly lower when associated with *D*. *perlucidum* (p = 0.0003) (Fig 3, Table 3). In many cases, the plant tissue had disintegrated within the *D*. *perlucidum* colony. Differences in leaf biomass loss were not significant across the 3 sampling seasons (p = 0.059), implying that loss of biomass due to smothering by *D*. *perlucidum* did not change seasonally. The significant difference between the sites could be attributed to the spatial and seasonal variability in *D*. *perlucidum* and *H*. *ovalis* abundance (p = 0.001).

**Table 3:** Analysis of Variance by Permutation (PERMANOVA) testing for differences in *H*. *ovalis* biomass with and without *D*. *perlucidum* (fixed factor), times of the year (fixed factor) and sites (random, nested in season). The analyses were based on Euclidean Distances calculated from Log(x+1) transformed data.

Source	Df	MS	Pseudo-F	P(perm)	Unique perms
**Treatment**	1	5.083	34.6	<0.001	9832
**Season**	2	6.497	3.84	0.060	9819
**Site(season)**	10	1.693	13.04	<0.001	9939
**Treatment x season**	2	1.081	7.36	0.012	9959
**Treatment x site**	9	0.147	1.13	0.362	9934
**Residual**	52	0.130			

**Fig 4:**
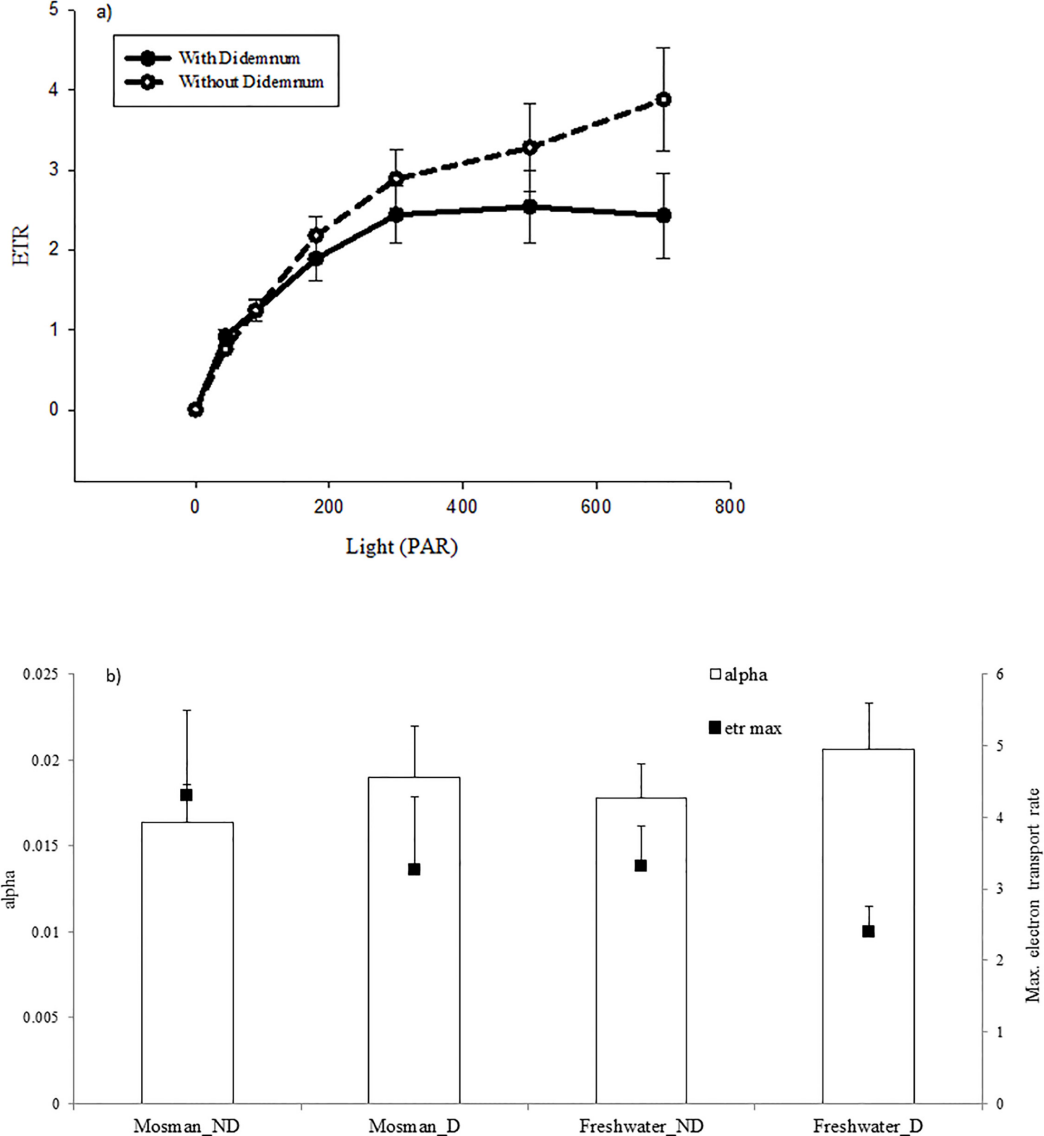
**a)** Relative light curve of *H*. *ovalis* tissues with and without *D*. *perlucidum*. **b)** α and ETR_max_ of *H*. *ovalis* tissue. D = plant tissue which had been covered with *D*. *perlucidum* colony. ND = plant tissue not impacted by *D*. *perlucidum*

Differences in photosynthetic responses were not significantly different between live Halophila leaves with and without fouling by D. perlucidum (Fig 4). The differences between the rapid light were not statistically significant and at both sites, there were no significant differences recorded for ETRmax or α (ETRmax p = 0.247, α p = 0.251). Differences in chlorophyll a pigments were also not significant between leaves with and without coverage of D. perlucidum (p = 0.259).

**Fig 5:**
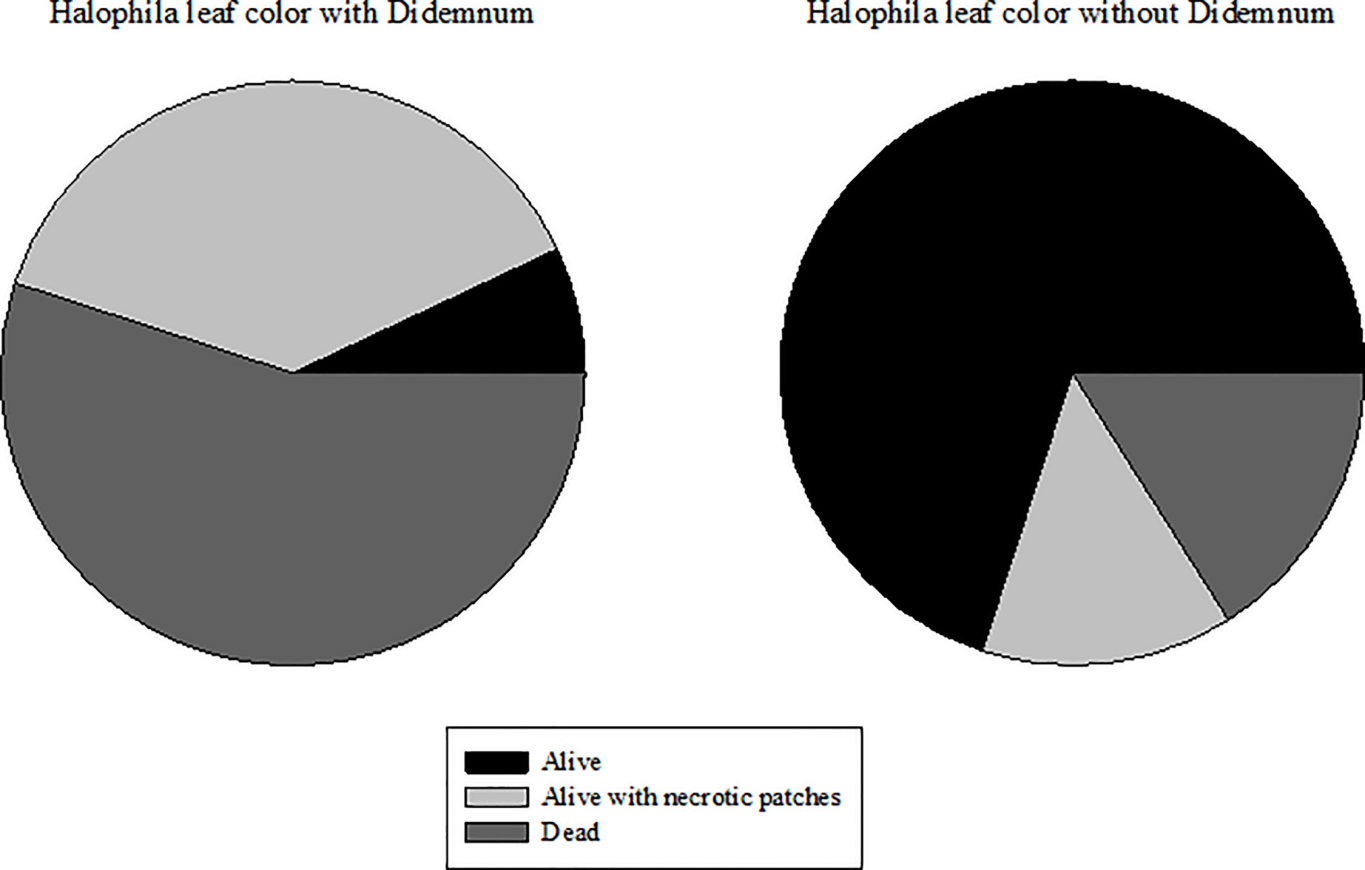
Relative health index of *H*. *ovalis* leaves with and without *D*. *perlucidum*. 1 = alive (green), 2 = alive with necrotic patches (green with brown spots), 3 = dead (various shades of brown).

Seagrass leaves within *D*. *perlucidum* colonies remained strongly attached to their stolons, which were rooted in the sediment. This suggests that leaves were alive when *D*. *perlucidum* settled and began to cover them rather than being dead or dying prior to settlement. This is further supported by the observation that *D*. *perlucidum* was seen growing on individual live leaves, and in sampling leaves around the *D*. *perlucidum* colonies it was evident that over 70% of the surrounding leaves appeared to be alive and healthy. The leaves chosen to measure photosynthesis and pigments were those which were still alive and photosynthetically active. As examined with the health index, this represented a small percentage of all the leaves within *D*. *perlucidum* colonies. Only 7% of the leaves within any given colony still maintained a bright green colour and only about 38% had some remaining green colour but were starting to turn brown, indicating that they were unhealthy or dying. The remaining 55% of leaves were various shades of brown suggesting they were already dead. This was compared to samples of unaffected leaves, in which more than 70% of leaves were green and alive (Fig 5). While photosynthetic rates and pigments of individual leaves might be marginally lower when associated with *D*. *perlucidum*, the measured leaves were part of a greater assemblage of mostly dead leaves. This suggests that measuring photosynthesis on the remaining live leaves may be underestimating the overall reduction in production capacity of *H*. *ovalis*. The lack of manipulation in the design of this study limits the ability to fully interpret these findings and used in isolation, fluorescence parameters are not a good indicator of stress in response to *D*. *perlucidum*.

### Effects on *Batillaria australis*

**Fig 6:**
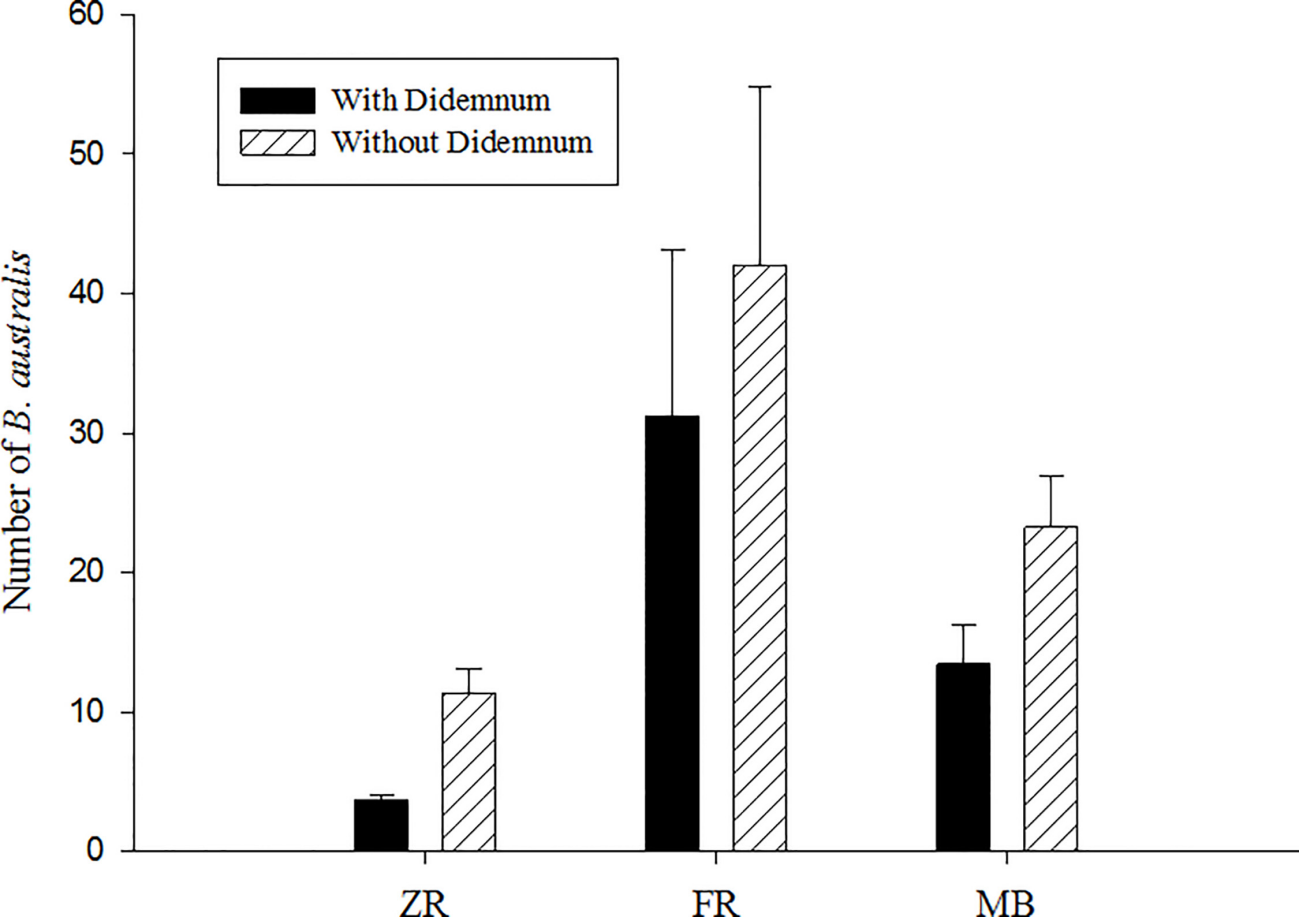
Counts of *Batillaria australis* with and without *D*. *perlucidum* colonies sampled from 9mm sediment cores and pooled from 3 sites in April and June 2014 and March 2015. (n = 9 with *D*. *perlucidum* per site and 9 without *D*. *perlucidum* per site).

There were significantly lower densities of *B*. *australis* in the presence of *D*. *perlucidum* associated with *H*. *ovalis* (p = 0.003). Average counts of *B*. *australis* were 1.3 to 2.8 times higher in cores without *D*. *perlucidum* than those with colonies present (Fig 6).

## Discussion

Globally there has been rapid, large scale seagrass loss associated with multiple stressors including climate change, shifts in water quality and localised impacts such as pollution and increased sediment and nutrients [2, 11, 12]. The introduction of invasive species, particularly ascidians, is another stressor contributing to seagrass loss [25, 26, 27]. This study has shown that in the Swan River, a typical urban estuary, the invasive colonial ascidian *Didemnum perlucidum* has a widespread seasonal coverage throughout the lower reaches of the estuary with the potential to directly contribute to loss of the native seagrass *Halophila ovalis*. As far as we are aware, this is the first time that this species has been documented fouling on natural substrata such as live seagrass. We have shown that it has a measurable effect on small scale patches of seagrass, impacting the biomass of leaves that it settles on and also influencing the abundance of associated fauna. While it is difficult to measure, the photosynthetic ability of leaved fouled by *D*. *perlucidum* may also be reduced. The presence of human infrastructure, particularly boat moorings, was found to be the best predictor of *D*. *perlucidum* distribution in the estuarine environment, suggesting these structures facilitate the spread to, and impact on, seagrass beds.

### Distribution of *Didemnum perlucidum*

In this study, the distribution of *Didemnum perlucidum* in the lower estuary of the Swan River was patchy and seasonal, and appeared to be driven by the presence of infrastructure and the hydrology of the river, particularly the changes in salinity.

The abundance of *D*. *perlucidum* colonies was consistently most prevalent in areas with artificial structure including boat moorings, jetties, yacht clubs and navigation markers. The first observations of colonies each year were concentrated around these structures (Simpson personal observation), suggesting they facilitated recruitment into seagrass beds. This also helps to explain the mechanism of dispersal and recolonization of *D*. *perlucidum* each summer. Settlement arrays in the inner harbour area of Fremantle Port (at the river mouth) and around nearby Garden Island have demonstrated overwintering colonies (Simpson unpublished data). It is possible that these colonies provide larvae for recruitment upriver as conditions become more favourable. There may also be overwintering colonies in deep pockets of the river where salinity remains high even during winter flooding, such as in Mosman Bay which consistently had the highest cover of *D*. *perlucidum*. It is likely that *D*. *perlucidum* maintains small colonies on mooring buoys. Recreational vessels are a likely vector for spread of propagules from these surviving colonies throughout the river.

In addition, rafting on plant material could be another transport vector explaining the reappearance and spread of *D*. *perlucidum*. Rafting is an important process for the population dynamics of many organisms including ascidians and it has a strong influence on coastal biodiversity. Rafting on eelgrass is a previously described method of transport and spread for other ascidian species including Botryllids and *Ciona intestinalis* and has been suggested as a method of spread for *Didemnum vexillum* [18, 27, 38,39]. *H*. *ovalis* is vulnerable to dislodgement as the leaves grow from a shallow rhizome with only small roots below ground as an anchor. As leaves become smothered by *D*. *perlucidum* the extra drag increases the likelihood that the rhizome will break or the plant will become uprooted. They roll along the substrate and when they get snagged may induce settlement in new areas. *D*. *perlucidum* is very capable of regenerating from fragmentation [20]. Colonies may overwinter in deep seagrass areas or on artificial substrate near the Port or on vessels but are able to spread quickly to other areas throughout the season through larval dispersal, fragmentation and/or rafting.

Hydrology of the river is also important in shaping the distribution of *D*. *perlucidum*. The Swan River is a microtidal estuary (tidal range <2m). Physical processes are driven by wind, wave action and freshwater runoff rather than tidal fluctuation. This results in a system of very low turbulence and high density stratification [40]. The hydrology of the Swan River changes seasonally due to the nature of winter rainfall and following catchment runoff [40, 41]. Early in the summer, a salt wedge propagates upstream roughly 60km from the river mouth. As river flow increases in winter, there is usually a period of stratification before the salt wedge is pushed back out toward the lower estuary. Salt water remains longer in the deeper areas until the freshwater influx from rain eventually flushes the estuary [40]. This pattern of salinity helps to explain why *D*. *perlucidum* is able to survive so far upstream during the summer. Colonies were present throughout much of the lower estuary following periods of warm water and high salinity. As the salt wedge retreated with the influx of rainwater runoff, colonies retracted or died. *D*. *perlucidum* died off early near Point Walter because it is an area strongly influenced by freshwater runoff with low salinity by June. Mosman Bay and Chidley Point maintained large colonies later in the year because they are deeper sites which still had high salinity under the freshwater lens. By August the saltwater had moved out of the estuary, resulting in a complete lack of visible *D*. *perlucidum* survival throughout the winter.

Salinity and temperature are important environmental factors influencing the distribution and recruitment of *D*. *perlucidum* [22, 42]. Growth and reproductive effort are highest throughout the warmest months and larvae production, recruitment and growth decline during the winter [20]. Current knowledge of temperature thresholds suggest it can survive between 15 and 30°C [16] and *in situ* experiments have shown that *D*. *perlucidum* increases in cover and biomass when water temperature is increased [43, 44]. *D*. *perlucidum* can reproduce throughout the year but there is a reduction in colony size and larvae density during the period of winter to spring [20, 22]. But despite reduced recruitment, in a marine environment, *D*. *perlucidum* is able to continue to survive and reproduce throughout the year [22, 42]. Within the Swan River, it is likely that the same pattern would persist. However, with the freshwater input through the estuary, the salinity becomes too low for ascidian survival and they appear to completely die off. In this study, our model suggested no evidence that temperature was a major driver in determining the distribution of *D*. *perlucidum*. This may be because although temperature is important to many aspects of the biology of *D*. *perlucidum*, salinity is most important in determining its survival. The salinity threshold has not yet been established but observations of *D*. *perlucidum* have shown a retraction of colonies in coastal environments during winter when freshwater runoff is increased, while colonies offshore do not seem to retract through winter (Simpson, personal observation). Multi-factorial studies need to be undertaken to determine the minimum salinity and temperature thresholds for survival and performance of this species.

### Effect on seagrass

Compared to other plant groups worldwide, seagrasses require very high light levels to provide oxygen to their roots and support large amounts of non-photosynthetic tissue [2]. This light requirement means that seagrasses are highly influenced by environmental changes that alter light levels reaching the plant, such as turbidity [2], shading and fouling [26]. Fouling of *D*. *perlucidum* on *Halophila* leaves appears to cause the leaves to die and decompose within the colony. Ascidians can reduce PAR between 10 and 95% depending on the morphology of the zooids [26]. *D*. *perlucidum* would be particularly variable because the presence of calcareous spicules in the tunic increases attenuation. The PAM analysis suggests that *D*. *perlucidum* is having an effect on the photosynthetic ability of the plant tissue but it cannot be concluded that reduced photosynthesis is the mechanism killing the plant.

*H*. *ovalis* has a low tolerance to light deprivation, with complete plant death occurring after 38 days in the dark [45]. As *D*. *perlucidum* can persist for several months, the shading induced by this ascidian could clearly deprive the seagrass of the light required for effective photosynthesis. With limited tolerance to light deprivation, the long-term survival strategy of this species may be based on its ability to rapidly regrow from seed and/or vegetative fragments after light deprivation [45]. If the long term survival strategy of *Halophila* is limited by other anthropogenic stressors, the additive effect of ascidian fouling will put additional pressure on the decline of seagrass meadows.

In 2011, it was estimated that the Swan River estuary supported 403 ha of *H*. *ovalis* [10]. In its peak season, as shown by measurements from April 2014 and March 2015, *D*. *perlucidum* was present at 75% of the observed sites throughout the estuary, with its mean abundance being 3% cover. That would hypothetically equate to 9ha of *H*. *ovalis* being smothered by *D*. *perlucidum* during the peak season. Given an average biomass loss of 43%, regardless of season, that implies that as much as 3.9 ha of *H*. *ovalis* could be lost every year during the peak *D*. *perlucidum* season, the same area as 6 soccer fields. During the low season, as measured in June 2014, 58% of the estuary had a *D*. *perlucidum* presence, with a mean cover of 2% at affected sites. This equates to 4.7 ha with a total potential loss of 2 ha of biomass. While this is a hypothetical extrapolation, it provides an estimation of the potential loss of *H*. *ovalis* at the estuary scale.

### Effects on *Batillaria australis*

Studies on community structure in marine systems have often shown that invading species have the potential to displace resident species [46, 47, 48] and in this study *Batillaria australis* was surveyed as an example of a benthic species that may be effected by the presence of an invasive fouling tunicate. This species was chosen as an example because it is the most abundant macroinvertebrate in the Swan River estuary [37]. It is also very important in creating habitat and facilitating overall diversity of benthic species [49]. There were significantly higher numbers of the mud snail *B*. *australis* in cores without *Didemnum perlucidum* colonies. Thomsen et al. [50] found the opposite response to be true when the macroalgae *Gracilaria comosa* covered *Halophila*. It created a ‘habitat cascade’ where habitat forming seagrass provided living space and protection for another habitat former (macroalgae) which then increased facilitation of invertebrate species including *B*. *australis*. However, where macroalgae provides oxygen, additional food and detritus used by the snails, *D*. *perlucidum* provides no nutritional benefit and at the same time seals off the decomposing seagrass within its acidic tunic. Didemnids have a pH within their tunic of less than 3, which is a level of acidity that deters most generalist fish predators [18, 51] other snails [27] and may deter *B*. *australis* as well.

In 2012 it was estimated that there were over 5.2 billion snails in the seagrass meadows of the Swan River estuary, which has a massive impact on moving sediment, releasing nitrogen, filtering water and producing shells as potential substrate [10, 37]. The peak abundance of *B*. *australis* also coincides with the peak abundance of *D*. *perlucidum*. Both species have the highest cover in the sites closest to the river mouth and both species appear to experience their highest recruitment during the late summer [10, 22]. *B*. *australis* populations also support other organisms such as billions of macroalgae attached to living snails and >100 million hermit crabs living in empty shells [37] as well as providing habitat to communities of sessile invertebrates [49]. Further research is needed to determine the extent of interaction between these two species. However, given the abundance, distribution and ecosystem function that this species already has, the interaction of high numbers of *D*. *perlucidum* with 5.2 billion snails could eventually cause ecosystem effects that would be very difficult to measure or predict.

## Conclusions

This study has demonstrated that *Didemnum perlucidum* is able to survive year after year colonizing native seagrass within an urban estuary. Its distribution is seasonal, patchy and largely driven by the presence of artificial infrastructure and changes in salinity. *D*. *perlucidum* has shown the potential to impact *Halophila ovalis* at the level of the individual plant through decreasing photosynthetic ability and loss of biomass. It has also shown the potential to interact with another species on a small scale.

Impacts from stresses on coastal marine communities are often manifested at the individual species level, but can magnify in effect throughout the entire ecosystem [13, 52]. Recent findings show that extremely consequential impacts at the ecosystem level (ie. trophic cascades, changing nutrient cycling, etc) may not be easily detected or may remain innocuous for some time [5] particularly in situations such as an estuary where population dynamics of an invader and the dynamics of the ecosystem vary over space and time. This demonstrates the need for regular long-term monitoring at an ecosystem level. The decline of seagrass is continuing in urban estuaries around the world due to many factors such as nutrient eutrophication, human impacts and increasing temperatures [2, 10, 12] and it is these stressful conditions that enable invasive ascidian populations to thrive [27]. The potential for impact from *D*. *perlucidum* or any other invasive ascidian species should not be ignored or underestimated.

## Supporting Information

S1 Fig Water conditions from Swan River during survey periods April and June 2014, and March 2015 listed from downstream to upstream. Conditions for August were obtained from the Swan River Trust weekly river profile reports
(TIF)

S1 Table Locations of observed *D*. *perlucidum* in the Swan River during the sampling periods of April and June 2014 and March 2015, listed from downstream to upstream (TIF)
