# Supplementary figures and images for: Distribution and Localised Effects of the Invasive Ascidian Didemnum perlucidum (Monniot 1983) in an Urban Estuary
Source: PLoS One. 2016 May 4;11(5):e0154201. doi: 10.1371/journal.pone.0154201 (PMC4856268; doi:10.1371/journal.pone.0154201)

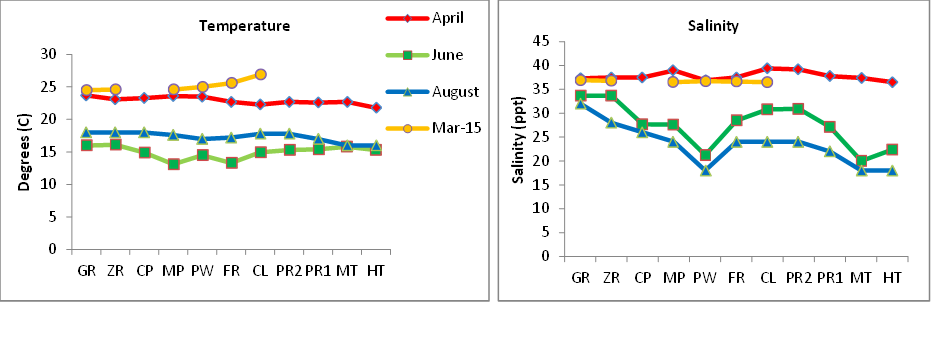

Supplement: S1 Fig — Conditions for August were obtained from the Swan River Trust weekly river profile reports (TIF) [file pone.0154201.s001.tif]

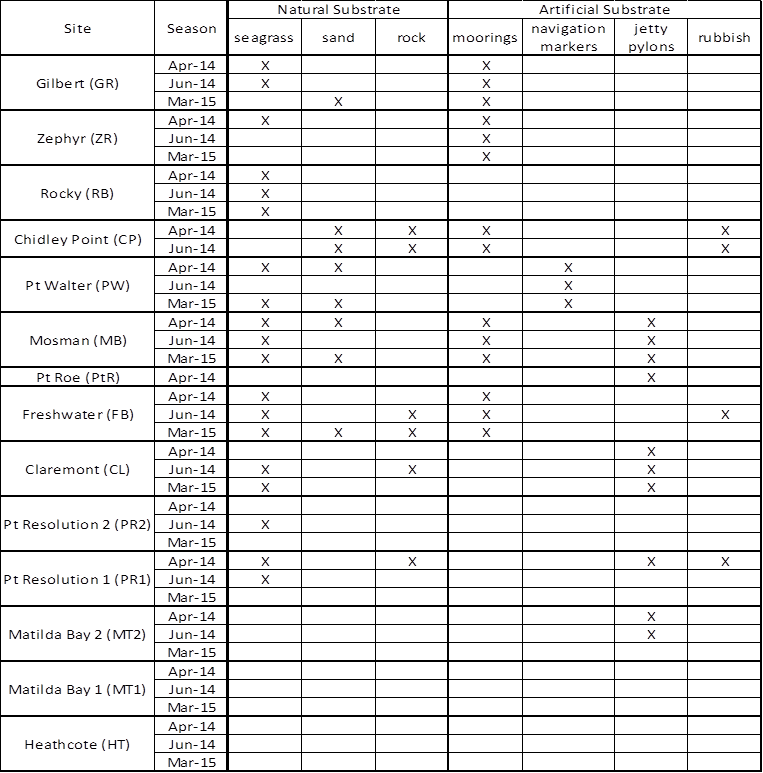

Supplement: S1 Table — (TIF) [file pone.0154201.s002.tif]
